# Nonuniform Late Pleistocene glacier fluctuations in tropical Eastern Africa

**DOI:** 10.1126/sciadv.abb6826

**Published:** 2021-03-12

**Authors:** Alexander R. Groos, Naki Akçar, Serdar Yesilyurt, Georg Miehe, Christof Vockenhuber, Heinz Veit

**Affiliations:** 1 Institute of Geography, University of Bern, Bern, Switzerland.; 2 Institute of Geological Sciences, University of Bern, Bern, Switzerland.; 3 Department of Geography, Ankara University, Ankara, Turkey.; 4 Faculty of Geography, Philipps University Marburg, Marburg, Germany.; 5 Laboratory of Ion Beam Physics, ETH Zurich, Zurich, Switzerland.

## Abstract

Today’s ice caps and glaciers in Africa are restricted to the highest peaks, but during the Pleistocene, several mountains on the continent were extensively glaciated. However, little is known about regional differences in the timing and extent of past glaciations and the impact of paleoclimatic changes on the afro-alpine environment and settlement history. Here, we present a glacial chronology for the Ethiopian Highlands in comparison with other East African Mountains. In the Ethiopian Highlands, glaciers reached their maximum 42 to 28 ka thousand years ago before the global Last Glacial Maximum. The local maximum was accompanied by a temperature depression of 4.4° to 6.0°C and a ~700-m downward shift of the afro-alpine vegetation belt, reshaping the human and natural habitats. The chronological comparison reveals that glaciers in Eastern Africa responded in a nonuniform way to past climatic changes, indicating a regionally varying influence of precipitation, temperature, and orography on paleoglacier dynamics.

## Teaser

Mountain glaciers in tropical Eastern Africa responded in a nonuniform way to Late Pleistocene climatic changes.

## INTRODUCTION

**Fig. 1:**
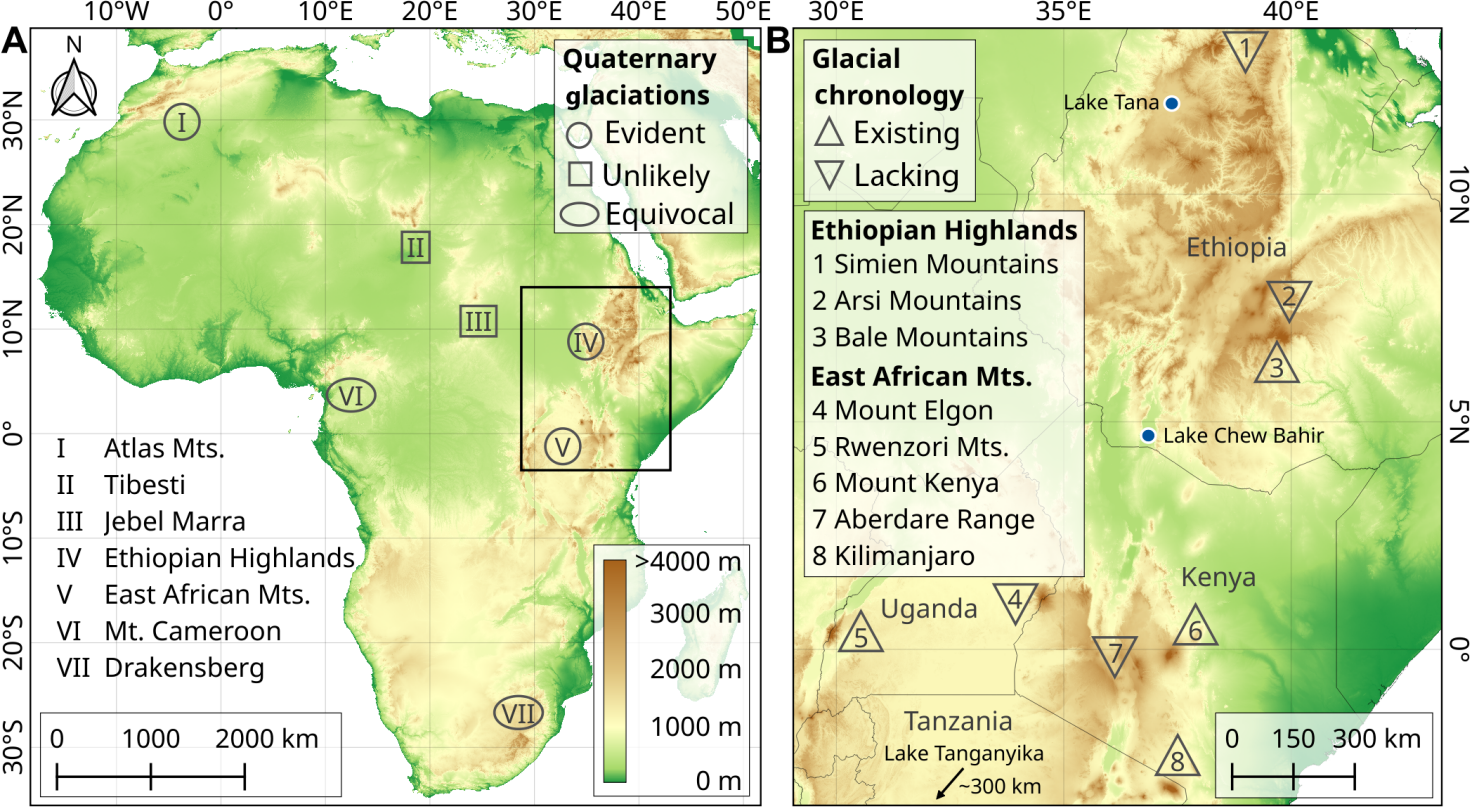
Quaternary glaciations in Africa. (**A**) Topographic map showing mountain localities with clear and controversial geomorphological evidence of past glaciations (*3*, *4*). (**B**) Overview of cosmogenic glacial chronologies from the Ethiopian Highlands and East African Mountains [sites IV and V in (A)] based on ^36^Cl and ^10^Be exposure ages (*7*, *9*–*12*). The blue circles and black arrow indicate the location of the paleoclimate archives (Lake Tana, Chew Bahir, and Tanganyika) considered for Fig. 6 and Discussion.

The extant glaciers in Africa are restricted to the summit areas of Mount Kenya, Kilimanjaro, and Rwenzori Mountains (*1*, *2*), but during the cold periods of the Late Pleistocene, several mountain ranges on the continent were extensively glaciated (table S1) (*3*–*5*). Well-preserved moraine sequences and other glacial landforms testify to multiple glacier advances in the High Atlas, East African Mountains, and Ethiopian Highlands (Fig. 1). Since past glacial fluctuations mainly reflect long-term changes in temperature, precipitation, cloudiness, and insolation, glacial landforms are an appropriate proxy for reconstructing regional paleoclimatic variations and paleoecological changes in alpine environments (*4*). Studying the climate and glacial history of the mountains in Eastern Africa is of particular interest since the topography on both sides of the East African Rift along with an amplified regional cooling at high elevations favored the formation of numerous ice caps and valley glaciers (*3*–*6*). Furthermore, assessing the impacts of past glaciations on the afro-alpine environment plays a key role in understanding the circumstances of the early migration of Middle Stone Age foragers into the high elevations of the glaciated Ethiopian Highlands 47 to 31 cal ka BP (calibrated kiloanni before present) (*7*). Information on paleoclimatic and paleoecological changes in the afro-alpine environment during the Pleistocene is also of high relevance for elucidating why tropical mountains are biodiversity hot spots (*8*). Latest glacial chronological and paleoclimatological studies from the East African Mountains and Ethiopian Highlands indicate distinct climatic and ecological changes at high elevations as they provide evidence for multiple glacier advances, a pronounced cooling, and depression of altitudinal vegetation belts during the Late Pleistocene (*7*, *9*–*12*).

A recent comparative study focusing on paleoglacier fluctuations in the Rwenzori Mountains (Eastern Africa) and Andes (South America) suggests that tropical glaciers reached their last glacial maxima at ~29 to 20 ka (kiloanni) and hypothesizes that high-latitude warming initiated the onset of deglaciation in the tropics at ~20 to 19 ka (*11*). However, first ^36^Cl surface exposure ages of erratic boulders from two moraine sequences in the Bale Mountains (Ethiopian Highlands) indicate a more complex and nonuniform response of tropical glaciers to Late Pleistocene climate changes (*7*). On the basis of existing data, the glaciers in the northwestern valleys of the Bale Mountains reached their local Last Glacial Maximum (LGM) (*7*) during marine isotope stage (MIS) 3 and, therefore, far earlier than in the Rwenzori Mountains during MIS 2 (*11*). A major glacier advance in the Bale Mountains during the hypothesized pantropical LGM (~29 to 20 ka) (*11*) has not yet been verified (*7*). The emerging discrepancy between the timing of the local LGM in the East African Mountains (1°N to 3°S) and more northern Ethiopian Highlands (7° to 13°N) may indicate a regionally varying influence of precipitation, temperature, and orography on past glacial fluctuations. To investigate possible regional differences in the timing and extent of past glaciations in Eastern Africa and to detect changes in large-scale tropical atmospheric circulation systems, a representative glacial chronology of the Ethiopian Highlands including direct moraine ages from valleys and mountains over a wide area is necessary.

Here, we present an extensive glacial chronology dataset for the Ethiopian Highlands based on 21 previously published (*7*) and 59 new ^36^Cl surface exposure ages of boulders from moraines and periglacial features from the Bale and nearby Arsi Mountains. By combining the new ^36^Cl ages from the Ethiopian Highlands with existing ^36^Cl and ^10^Be glacial chronologies from Mount Kenya, Kilimanjaro, and the Rwenzori Mountains (*9*–*12*), this study aims to investigate the response of tropical glaciers in Eastern Africa to Late Pleistocene climate changes and elaborate on the paleoclimatic and paleoecological implications of past glacial fluctuations. Since the extent and dynamic of past glaciations are determined not only by the prevailing climatic conditions but also by orography and the potential surface area above the former equilibrium line altitude (ELA) (*13*), this study also includes the first terrain analysis of the most extensively glaciated African mountains (*3*). Considering the varying hypsography of the mountains is crucial for comparing and interpreting regional differences of past glacial fluctuations and drawing conclusions about the paleoclimate.

## RESULTS

### Hypsography of Africa’s glaciated mountains

Africa’s high mountains cover less than 0.1% of the entire area of the continent and are located mainly along the East African Rift (Fig. 1). Exceptions are the High Atlas in northwestern Africa, Mount Cameroon in western Africa, and the Drakensberg in southern Africa as well as the Tibesti in the Sahara and Jebel Marra in the Sahel. The afro-alpine area above 3500 m equals 0.016% (5041 km^2^) and that above 4000 m is 0.0028% (880 km^2^) of the continent’s total area of 31.6 million km^2^.

**Fig. 2:**
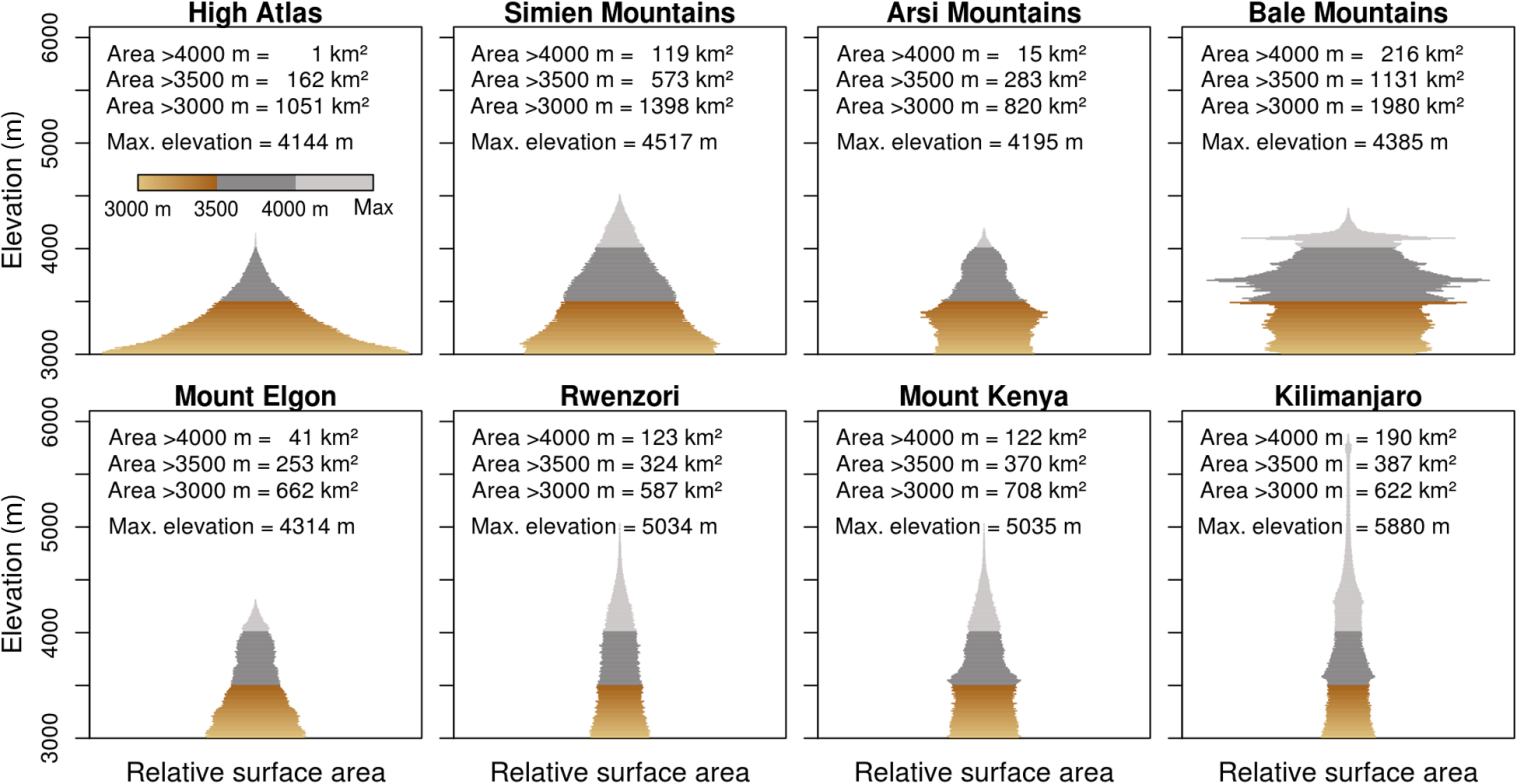
Hypsography of the eight African mountains that were most extensively glaciated during the Late Pleistocene. Top row (excluding High Atlas) shows the hypsography of the three highest mountains in the Ethiopian Highlands and bottom row shows the hypsography of four East African mountains. Note that the total surface area of the Bale Mountains above 3000 m is more than twice as large as that of most of the other peaks.

The extent, elevation range, and hypsography of the Ethiopian Highlands, East African Mountains, and High Atlas in northern Africa differ widely (Fig. 2). The Ethiopian Highlands (Arsi, Bale, and Simien Mountains) reach maximum elevations between 4200 and 4500 m. In contrast, the highest East African Mountains (Kilimanjaro, Mount Kenya, and Rwenzori) rise above 5000 m and are defined by their topographic prominence and smaller area-elevation ratio. Africa’s highest mountain, Kilimanjaro, reaches almost 5900 m and covers an area of 387 km^2^ for elevations above 3500 m and 190 km^2^ above 4000 m. Mount Kenya and the Rwenzori Mountains also cover an area of more than 320 km^2^ above 3500 m and 120 km^2^ above 4000 m. Although the Ethiopian Highlands are lower in elevation, they comprise the largest part in total surface area of Africa’s alpine environment above 3000, 3500, and 4000 m due to the broad base of the mountains (Fig. 2). The hypsography of the Bale Mountains is exceptional because extensive subhorizontal plateaus define the different elevation bands and its surface area does not decrease evenly with elevation (Fig. 2). The broad base and extent of its central plateau places the Bale Mountains as Africa’s largest solitary alpine environment. They cover 8% of the continent’s area above 3000 m, 22% of the area above 3500 m, and 25% of the area above 4000 m. In contrast, the High Atlas is characterized by a broad base and exponential decrease of surface area with elevation. While 1051 km^2^ of the mountain range is located above 3000 m and 162 km^2^ above 3500 m, only 1 km^2^ exceeds 4000 m.

The varying hypsography of the mountains has implications for present and past glaciations on the continent. Under present climatic conditions, the ELA is located far above the maximum elevation of most of the African mountains. Glaciers can therefore only persist in the summit areas of the three highest peaks of the East African Mountains. However, once the ELA decreases below 4000 to 4500 m as during the last glaciation (*3*), the potential surface area in Africa for the accumulation of snow and formation of ice increases drastically. While the entire area above 5000 m on the continent is limited to 22 km^2^ and the area between 4500 and 5000 m is limited to 61 km^2^, 796 km^2^ is available for snow accumulation between 4000 and 4500 m and an additional 4161 km^2^ is available between 3500 and 4000 m.

### Glacial history of the Bale Mountains

**Fig. 3:**
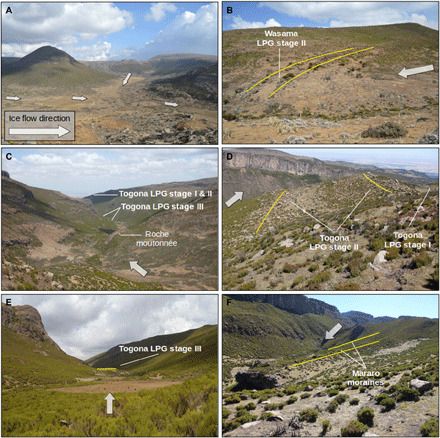
Glacial geomorphology of the northern valleys. White arrows indicate the approximate flow direction of the paleo valley glaciers. Yellow lines indicate preserved moraines. (**A**) View into the former accumulation basin of the Wasama Valley (northern margin of the plateau in the background). (**B**) Terminal moraine in the Wasama Valley. (**C**) Moraine sequence and roche moutonnée in the Togona Valley (view looking north). (**D**) Lateral moraines in the Togona Valley. (**E**) Innermost terminal moraine in the Togona Valley. (**F**) Lateral moraines in the Mararo Valley. Photo credit: Alexander R. Groos, Institute of Geography, University of Bern.

The central Sanetti Plateau above 3800 m in the Bale Mountains hosts several glacial and periglacial features (Fig. 3 and figs. S1 and S2). Large boulders (up to 8 m wide and 5 m high) encircle the highest peak (Tullu Dimtu, 4377 m) in the central part of the plateau at a distance of about 2.5 and 5 km from the peak (fig. S2, A and B). Because of their rounded shape and circular distribution around the peak, it is likely that a former ice cap transported the boulders and deposited them along its ice margins. The scatter of the erratic boulders on the plateau has previously been defined as the Big Boulder Moraine (*14*). Outside the Big Boulder Moraine, erratic boulders are rare. Subglacial till and small depressions resulting from irregular glacial erosion are typical for the northern plateau. The small depressions are seasonally filled with water and desiccate during the dry season (fig. S2E).

Large sorted stone stripes that are up to 2 m deep, 15 m wide, and 200 m long (fig. S2, C and D) are an outstanding geomorphological feature on the plateau. They are located on gentle slopes (4° to 8°) of two volcanic plugs about 3 and 5 km south of Tullu Dimtu (fig. S3, A and B) and in the far west of the plateau (fig. S3C). Sorted stone stripes of similar size are only known from periglacial environments in the mid and high latitudes, and their genesis presumably requires permafrost, an active layer and cyclic freezing and thawing (*15*, *16*). The hardly weathered surfaces suggest a rather young age for the formation of these features (e.g., global LGM or postglacial). Relict block fields characterize the upper part of the southern and southwestern escarpment (fig. S2F). In contrast to the western, northern, and eastern valleys, no moraines or any other glacial features were detected in the field or on high-resolution satellite images along the southern escarpment.

In the western, northern, and eastern U-shaped outlet valleys from the plateau, moraines and other glacial features such as roche moutonnées are well preserved (Fig. 3). We recorded numerous glacial features in several valleys of the Bale Mountains that were unexplored before. The terminal and lateral moraines in the valleys are mainly located between 3600 and 3900 m and suggest that the glaciers in the northwest were mainly 4 to 5 km and those in the northeast were up to 7 to 8 km long. The maximum ice thickness inferred from the elevation difference between the valley floors and preserved lateral moraines was about 200 to 300 m. Moraines consisting of fine and coarse material are rare in the Bale Mountains (fig. S1). Moraines made up solely of large trachytic and basaltic boulders (for the lithology, see fig. S4) prevail. The innermost moraines in the Wasama and Togona Valley are an exception. They are formed of unsorted glacial debris and are several meters high. The lowermost glacial features are weathered boulders that are distributed unevenly between 3500 and 3550 m in the plains of the northwestern Web Valley.

**Fig. 4:**
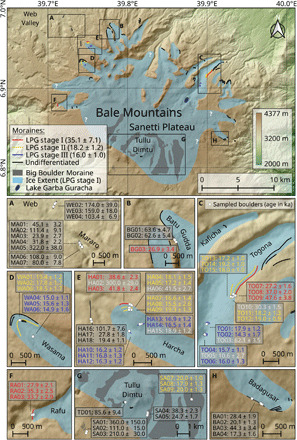
Glaciation map and ^36^Cl glacial chronology of the Bale Mountains. Presumed ice extent during LPG stage I. White question marks indicate tentative ice margins that are fraught with uncertainty due to the absence of direct geomorphological evidence. One white dot may represent multiple samples due to scale. The ^36^Cl ages are given in kiloanni (ka) with total uncertainties (1σ), are non-erosion–corrected, and were calculated using the web calculator of Marrero *et al*. (*17*) with its default production rates and the LSDn scaling scheme (*58*). Samples HA01 to HA15 and WA01 to WA06 from Ossendorf *et al*. (*7*) were recalculated using the same scaling scheme. Ages we consider as outliers are shown in white. The age of the LPG stages is the arithmetic mean (and the error is the SD, 1σ) of the population of selected boulders from moraines associated to that glacial stage (see table S7).

The 68 ^36^Cl surface exposure ages of moraine boulders from the Bale Mountains reveal a consistent glacial chronology for the U-shaped valleys and provide first insights into the deglaciation history of the paleo ice cap on Tullu Dimtu (Fig. 4). All exposure ages presented hereafter are non-erosion–corrected and calculated using the web calculator of Marrero *et al*. (*17*) with its default production rates and the time-dependent Lifton-Sato-Dunai (LSDn) scaling that accounts for geomagnetic field fluctuations and solar modulation (see Materials and Methods and tables S2 to S11). Along with the age, total uncertainty (1σ) is provided to enable the direct comparison with glacial chronological data and climate records from other regions. The choice of scaling causes considerable variability in tropical ^36^Cl production rates and exposure age calculation (0 to 10% for MIS 1 to 3 ages and 10 to 20% for MIS 4 and older ages in the Ethiopian Highlands; see table S6). However, since all exposure ages (except those from Mount Kenya and Kilimanjaro) were (re)calculated with the same time-dependent scaling, these uncertainties do not affect our major conclusions. We assume that the obtained exposure ages of a sampled moraine represent the stabilization phase of this landform and therefore mark the onset of glacial recession, which, in turn, indicates a pronounced change in glacier mass balance caused predominantly by an increase in temperature or decrease in precipitation. A large age scatter is certainly due to nuclide inheritance from earlier exposure periods (leading to age overestimation) or postdepositional processes such as toppling (leading to age underestimation) but may also be indicative of a compositional moraine from different glacial events or of a moraine that formed over a longer quasi-stable climate period. The stated age of a glacial event is provided as the arithmetic mean and the uncertainty as the SD (1σ) of boulders (excluding outliers) from landforms associated with that event.

The lowest erratic boulders in the Web Valley and three of the six moraine boulders from the Mararo Valley reveal exposure ages of more than 100 ka (Fig. 4) and could be interpreted in favor of a maximum ice expansion during MIS 6. However, the origin of the oldest landforms could also be MIS 4 or 3 if the ages greater than 100 ka are discarded as outliers. As the initial glaciation eroded old geological surfaces, substantial nuclide inheritance in the oldest dated boulders is very likely, more than during later glacial events. Nuclide inheritance along with postdepositional processes such as toppling, exhumation, and erosion probably explain the large age scatter (322 to 24 ka). On the basis of exposure ages of terminal and lateral moraines from the Rafu, Harcha, and Togona valleys in the Bale Mountains (Fig. 4), the local LGM is dated to the period ~42 to 28 ka (Late Pleistocene glaciation, LPG; stage I, 35.1 ± 7.1 ka, *n* = 9, outliers = 1). The age scatter may indicate either a longer persistent glaciation period or multiple glacier advances with a similar extent during MIS 3. Characteristic for the Batu Gudda Valley are two parallel lateral moraines (not visible in Fig. 4B due to scale; see fig. S1). The sampled boulder from the lower moraine relates to LPG stage I, but the two boulders from the upper lateral moraine are older and both consistently dated to ~60 ka. However, other landforms that clearly support a MIS 4 advance in the region have not yet been determined. In the other investigated northwestern and northeastern valleys (Wasama, Harcha, and Togona), a sequence of well-preserved moraines indicates two successive glacier culminations between 19 and 17 ka (LPG stage II, yellow, Fig. 4; 18.2 ± 1.2 ka, *n* = 16, outliers = 2). The ice extent during these LPG stage II culminations was similar to that during the local LGM. Deglaciation in the Bale Mountains began around 18 to 17 ka. The innermost terminal moraines in the same three valleys probably originate from a glacier stillstand dated to ~16 ka (LPG stage III, blue, Fig. 4; 16.0 ± 1.0, *n* = 12, outliers = 3). The pronounced lateral moraine in the Badagusar Valley appears to be a composite moraine comprising ages from all three LPG stages (44 to 17 ka). A tight sequence of multiple terminal moraines mapped downvalley of the sampled spots seems to be connected to the lateral moraine. This is a further indication that glaciers in the valleys of the Bale Mountains reached a similar extent multiple times during MIS 3 and 2. The element composition of boulders from the different valleys and glacial stages in the Bale Mountains varies considerably (fig. S4). While moraine boulders from valleys, which were probably decoupled from the plateau glaciation (e.g., Harcha), share the same lithology, the lithology of the LPG stage I and II and stage III moraines in the Togona Valley, which hosted the largest outlet glacier from the plateau during LPG stage I (Fig. 4), differs. The different lithologies (fig. S4) suggest that the moraine boulders of different stages in this valley originate from different catchments on the plateau, possibly because of a varying ice cap geometry over time.

In contrast to the valleys, the glacial chronology of the central Sanetti Plateau is less understood. Exposure ages older than 100 ka were obtained for a cluster of boulders on the southern edge of the plateau. These boulders might support the hypothesis of an early maximum glaciation during MIS 6, but here, the same dating uncertainties apply as those discussed for the old landforms in the valleys. During LPG stage I, the plateau glaciation extended down into the western, northern, and eastern valleys (Fig. 4). Determining the local LGM ice cap extent was not possible because suitable boulders along the assumed former ice margin have not yet been investigated. The outer Big Boulder Moraine, encircling the highest peak Tullu Dimtu at a distance of ~5 km, was dated to ~20 to 18 ka and, thus, relates to LPG stage II in the valleys. However, it is unclear whether the ice cap and valley glaciers were still connected at that time. The two sampled boulders from the inner Big Boulder Moraine and the single sample closer to the summit, which must be chronostratigraphically younger, date to 24.7 ± 1.7, 38.3 ± 2.3, and 85.6 ± 9.4 ka, respectively, and are not in agreement with LPG stage III in the valleys. They are considered to overestimate the deposition age of boulders due to nuclide inheritance and insufficient surface erosion during the short transport (<500 m) of the boulders downslope of Tullu Dimtu. How long the isolated ice cap persisted on Tullu Dimtu remains unspecified.

The exposure ages obtained from rock samples of the sorted stone stripes scatter and do not reflect a global LGM or postglacial formation age as suggested by the hardly weathered surface of the columnar basalt and trachyte (fig. S1). Three samples from the stone stripes south of Tullu Dimtu reveal ages of 200 to 67 ka. Even older ages (790 to 406 ka) were obtained for the western stone stripes (fig. S3). The very old ages and their excessive age range indicate that despite presumably ongoing periglacial activity during the last glacial cycle, dating these permafrost features on the Sanetti Plateau is problematic since the rocks must have experienced a long-term pre-exposure before the formation of the stone stripes. Moreover, the high ^36^Cl concentrations and well-preserved structure plus the absence of any boulders or glacial landforms nearby suggest that this part of the plateau was never covered by thick ice during the recent past (i.e., Late Pleistocene). Hence, the stone stripes serve as evidence for the southern and western maximum limits of the plateau glaciation during LPG stage I.

During LPG stage I, the extent of the former ice cap on Tullu Dimtu with respect to the limits of the outer Big Boulder Moraine was in the order of 75 km^2^. However, the topographic depressions across the northern plateau and smooth geomorphological transition of the plateau into the valleys suggest that the ice cap extended beyond the limits of the Big Boulder Moraine at that time and covered up to 155 km^2^ of the plateau. The western, northern, and eastern valley glaciers contributed another 110 km^2^ of ice coverage. Together, about 265 km^2^ (23% of the area above 3500 m) of the Bale Mountains might have been glaciated during LPG stage I (Fig. 4).

### Glacial history of the Arsi Mountains

Another Ethiopian mountain range that was extensively glaciated during the Pleistocene are the 4195-m-high Arsi Mountains, located east of the Main Ethiopian Rift and 100 km northwest of the Bale Mountains (Fig. 1). A large alpine plateau, which could hold an extensive ice cap similar to the one in the Bale Mountains, does not exist here (Fig. 2). Characteristic for the Arsi Mountains is the elongated relief and preservation of lateral and terminal moraines in most of the U-shaped valleys emanating out from the north-south trending ridge (fig. S5). The terminal and lateral moraines suggest that the valley glaciers were mainly between 2.5 and 5.5 km long and up to 200 m thick. Some of the moraine sequences comprise multiple glacial stages. The lowermost moraine in the investigated southwestern Garemba Valley covers an age range of 127 to 29 ka. The second innermost moraine is dated to 18.8 ± 0.4 ka (*n* = 2, outlier = 1) and correlates with the LPG stage II in the Bale Mountains. Three additional moraine ridges are located between LPG stage II and the maximum extent (fig. S5). One of these moraines may be associated with LPG stage I (see Materials and Methods). At that time, about 83 km^2^ (29% of the area >3500 m) of the Arsi Mountains were glaciated.

### Reconstructed ELAs and temperature depression

The LPG stage I moraines in the western and northwestern valleys of the Bale Mountains are mainly located between 3800 and 3850 m. In the northern and northeastern valleys, the glaciers extended further down and reached elevations below 3500 to 3700 m. The highest peaks at the glacier headwalls along the northern declivity exceed 4100 to 4300 m. This translates into a relatively small vertical extent of the valley glaciers in the order of 300 to 800 m. A terminus-to-headwall altitude ratio (THAR) of 0.5 revealed an LPG stage I ELA for the northwestern glaciers of 3940 ± 40 m (ELA_Harcha_ = 3980 m, ELA_Batu Gudda west_ = 3940 m, ELA_Batu Gudda east_ = 3900 m). These glaciers did not gain any mass input from the central ice cap and are therefore suitable for ELA reconstructions. The maximum elevation of lateral moraines (MELM) in the Harcha (3950 m), eastern Batu Gudda (3880 m), and Togona Valley (3800 m) as well as remains of a former cirque glacier in the Togona Valley (3850 to 3900 m) provide minimum values for the ELA and, thus, support the THAR results. The present annual 0°C isotherm is located at ca. 4650 m (fig. S6), ~270 m above the highest peak (Tullu Dimtu), and serves as an estimate for a theoretical modern ELA (see Materials and Methods). The difference between the theoretical modern ELA and the paleo ELA yields an ELA lowering of 710 m. Applying an estimated paleo lapse rate in the order of 7.5° ± 0.5°C km^−1^ (see Materials and Methods) to the calculated ELA lowering results in a temperature depression for the Bale Mountains of 5.3° ± 0.7°C at LPG stage I.

A mean LPG stage I ELA of 3840 ± 80 m was calculated for the neighboring Arsi Mountains using the THAR method. However, the individual ELAs of the west- and east-facing paleoglaciers differ widely (fig. S7). The average ELA of the east-facing glaciers (3780 ± 30 m) was about 125 m lower than that of the west-facing glaciers (3905 ± 35 m). This pattern is also supported by the lower glacial limits (3400 to 3500 m versus 3650 to 3750 m) and lower MELM in the eastern valleys (3800 versus 3900 m). The reconstructed ELAs from the Arsi and Bale Mountains agree within the margin of uncertainty and demonstrate that glacial geomorphological features in the Ethiopian Highlands are a valuable proxy for assessing regional paleoclimatic changes.

### Late Pleistocene glacial fluctuations in Eastern Africa

**Fig. 5:**
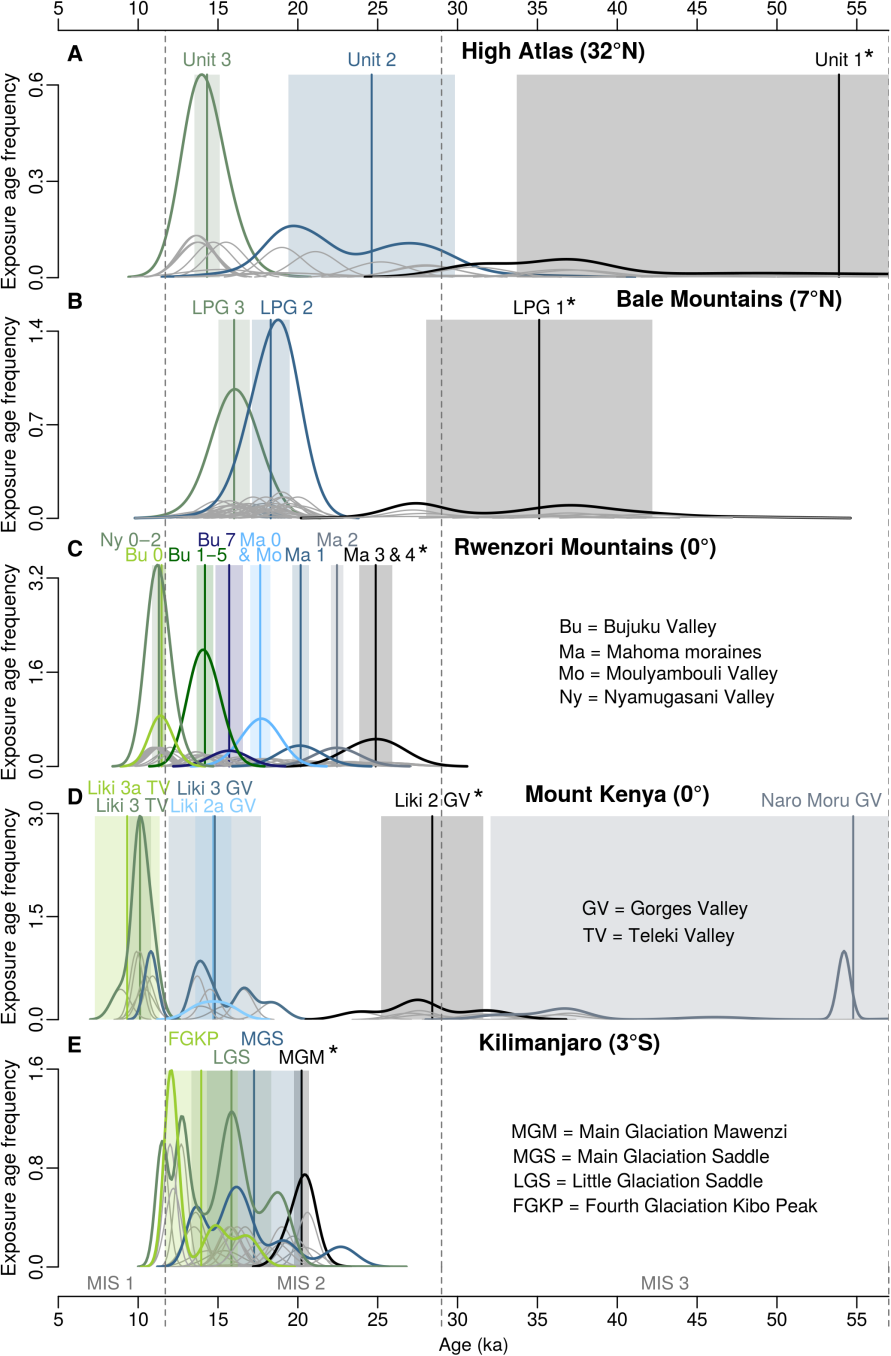
Late Pleistocene (MIS 1 to 3) glacier fluctuations in tropical Eastern Africa and the High Atlas. Normalized frequency distribution (normal kernel density estimate) of surface exposure ages of moraine boulders from (**A**) the High Atlas (^36^Cl and ^10^Be) as an example of an extratropical glacial system (*19*), (**B**) Bale Mountains (^36^Cl) [(*7*) and this study], (**C**) Rwenzori Mountains (^10^Be) (*10*–*12*), (**D**) Mount Kenya (^36^Cl) (*9*), and (**E**) Kilimanjaro (^36^Cl) (*9*). Each gray Gaussian curve illustrates the age (with a total error of 1σ) of one sample. The flatter the curve, the larger the uncertainty. Bold black, blue, and green curves represent the sum of all exposure ages (i.e., curves) from the same glacial stage (excluding outliers) as defined in the original publications (see Materials and Methods and tables S8 to S11) (*7*, *9*–*12*, *19*). The flatter the bold curve, the smaller the sample size and the larger the uncertainty of a glacial stage. The straight vertical lines and shaded areas display the arithmetic mean and uncertainty (1σ), respectively, of each glacial stage. The local LGM of each region is marked with an asterisk. The dashed vertical lines are the MIS boundaries. Note that the exposure ages from Mount Kenya and Kilimanjaro could not be recalculated using updated production rates and a time-variant scaling. Direct comparisons between both chronologies and the rest of the data may not be valid.

The combined analysis of the glacial chronology from the Ethiopian Highlands and previously published chronologies from the Rwenzori Mountains (*10*–*12*), Mount Kenya, and Kilimanjaro (*9*) reveals that tropical glaciers in Eastern Africa responded in a nonuniform way to climatic changes during MIS 3 and 2 (Fig. 5). In the southern Ethiopian Highlands, the local LGM (LPG stage I) occurred already between ~42 and 28 ka (MIS 3), far before the global LGM [22.1 ± 4.3 ka; after (*18*)]. A similar pattern is also evident from the extratropical High Atlas in northwestern Africa, where glaciers reached their maximum expansion (glacial unit 1) in the period ~75 to 35 ka (*19*) and, thus, also before the global LGM. On Mount Kenya, the presumed local LGM was dated to 28 ± 3 ka (Liki II moraine in the Gorges Valley), and on Kilimanjaro, it was dated to 20 ± 1 ka (Mawenzi main glaciation) (*9*). However, ages of individual glacial events on Mount Kenya and Kilimanjaro must be interpreted cautiously, as a recalculation with updated production rates and a time-dependent scaling was not possible (see Materials and Methods). This hinders the comparison with other glacial chronologies and paleoclimate proxies. A consistent glacial chronology from the lower Mubuku Valley in the Rwenzori Mountains testifies to major extents at ~26 to 25 ka (Mahoma stage 3 and 4), ~23 to 22 ka (Mahoma stage 2), and ~20 to 19 ka (Mahoma stage 1) during the global LGM (*10*, *11*). The main MIS 3 and 2 glacial events in the Ethiopian Highlands (LPG stage I and II) occurred rather at the beginning and end (Fig. 5) than during the hypothesized pantropical LGM [~29 to 20 ka after (*11*)]. A major advance in the Ethiopian Highlands during this period seems unlikely with respect to the available data. A distinct glacial stage that is clearly recognizable in the Ethiopian Highlands (LPG stage II) as well as in the Rwenzori Mountains (Moulyambouli and Mahoma 0) is dated to ~19 to 17 ka (Fig. 5). However, while the ice extent in the Harcha and Togona Valley in the Ethiopian Highlands during LPG stage II is similar to the local LGM extent (i.e., LPG stage I), the Mahoma 0 moraine in the Rwenzori Mountains is located almost 5 km up the valley from the Mahoma 1 terminus (*11*). Despite spatiotemporal differences of the major glacial stages in Eastern Africa and the High Atlas, distinct glacier shrinkage after ~16 to 12 ka is verified for all studied African mountains at the end of the last glacial cycle (Fig. 5) (*7*, *9*, *12*, *19*). In the Bale Mountains, the certainly last glacial stillstand at 16.0 ± 1.0 ka is in line with the basal ages of a sediment core from the cirque lake Garba Guracha (3940 m), which is located in the head of the Togona Valley (Fig. 4). The basal ages of the core suggest that sedimentation in the Togona Valley started after ~16 cal ka BP (*20*). This leads to the conclusion that the outlet glacier in this valley retreated rapidly from its LPG stage III position. The valley became probably ice free within a few decades or centuries after LPG stage III.

Not only the timing but also the reconstructed maximum ice extent and percentage of the glaciated area above the lower ice limits (~3500 m) (*3*) during the last glacial cycle differ widely between the individual mountains in Eastern Africa. The four most extensive Late Pleistocene glaciations occurred in the Bale Mountains, in the Rwenzori Mountains, and on Mount Kenya and Kilimanjaro (table S1). This observation is in agreement with the mountain’s high maximum elevation and large area above the paleo ELA (Fig. 2). When comparing the percentage of the glaciated area above the lower ice limits of the individual mountains, a distinct spatial pattern becomes apparent: The percentage of the glaciated area above 3500 m is much higher in the East African Mountains near the equator (Rwenzori = 62 to 80%, Mount Kenya = 54 to 65%, Kilimanjaro = 39 to 52%, and Mount Elgon = 30 to 38%) than in the more northern Ethiopian Highlands (Arsi = 29%, Bale = 23%, and Simien = 2%).

## DISCUSSION

**Fig. 6:**
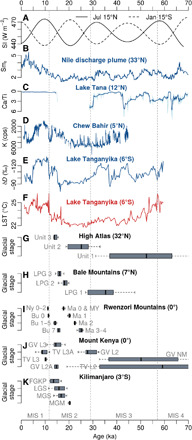
Comparison of different climate proxies and glacial chronologies from Eastern Africa and the High Atlas for the last 71 ka (MIS 1 to 4). (**A**) Solar irradiance (SI) variations at 15°N (July) and 15°S (January) (*66*). (**B**) Sm_r_ record (smectite to illitite + chlorite ratio) from the Nile discharge plume off Israel indicating wet (↑) and dry (↓) phases in the northern Ethiopian Highlands (*29*). (**C**) Ca/Ti record (calcium-to-titanium ratio) from Lake Tana (northern Ethiopian Highlands; see Fig. 1B) indicating high (↑) and low (↓) lake levels (*31*). (**D**) Potassium record (K in counts per second) from Chew Bahir (Southern Ethiopian Rift; see Fig. 1B) indicating wet (↑) and dry (↓) periods (*30*). (**E**) Deuterium record from Lake Tanganyika (East African Rift; see Fig. 1B) indicating wet (↑) and dry (↓) periods (*26*). (**F**) Lake surface temperature (LST) proxy from Lake Tanganyika (*26*). (**G** to **K**) Boxplots showing the exposure age distribution (minimum, maximum, median, and interquartile range) of Late Pleistocene glacial stages in the High Atlas (*19*), Bale Mountains [(*7*) and this study], Rwenzori Mountains (*10*–*12*), Mount Kenya (*9*), and Kilimanjaro (*9*). For more details on the applied cosmogenic nuclides and acronym explanations, see Fig. 5. Note that the exposure ages from Mount Kenya and Kilimanjaro could not be recalculated using updated production rates and a time-variant scaling. Direct comparisons between both chronologies and the rest of the data may not be valid.

Comprehensive glacial geomorphological and chronological investigations from the Bale and Arsi Mountains presented here demonstrate that extensive plateau and valley glaciers formed in the southern Ethiopian Highlands during the Mid and Late Pleistocene. ^36^Cl surface exposure ages of moraine boulders provide evidence that glaciers in the Ethiopian Highlands reached their local LGM (LPG stage I) in the period ~42 to 28 ka, well before the global LGM (*18*). Ice covered about 265 km^2^ of the Bale Mountains (Fig. 4) and 83 km^2^ of the neighboring Arsi Mountains (fig. S5) at that time. The maximum glacier extent was accompanied by a mean temperature depression in the region of 5.3° ± 0.7°C. A sequence of post-LGM glacier culminations was dated to ~19 to 17 ka (LPG stage II), the onset of deglaciation was dated to ~18 to 17 ka, and a last glacial stillstand was dated to ~16 ka (LPG stage III). The comparison of the ^36^Cl glacial chronology from the southern Ethiopian Highlands with previously published ^36^Cl and ^10^Be glacial chronologies from Kilimanjaro, Mount Kenya, and the Rwenzori Mountains highlights regional variations in the response of tropical glaciers in Eastern Africa to Late Pleistocene climate changes (see Figs. 5 and 6). This finding challenges the persisting idea of rather uniform glacier expansion and shrinkage in the tropics during the Late Pleistocene (*10*–*12*).

On the basis of the latest reevaluation of published ^10^Be moraine ages from the Andes and new ^10^Be data from the Rwenzori Mountains, Jackson *et al*. (*11*) concluded that tropical glaciers reached their last glacial maxima in the period ~29 to 20 ka and started to recede at ~20 ka. The authors hypothesize that their observed “early” onset of deglaciation in the tropics before the rapid CO_2_ rise at ~18 to 17 ka was due to high-latitude warming, which reduced the thermal gradient between the polar regions and tropics and decreased the heat removal from the tropics toward the high latitudes. A key assumption underlying this hypothesis is that tropical glaciers are most sensitive to changes in temperature. As a modern analogy and evidence that temperature is the main climatic control of glacier mass balance in the humid inner tropics of Eastern Africa, Jackson *et al*. (*11*) refer to a study by Taylor *et al*. (*21*), which claims that rising temperatures are the dominant factor for recent glacier melting in the Rwenzori Mountains. However, the detailed comment on this study by Mölg *et al*. (*22*) elaborating on the importance of other climate variables for the energy and mass balance of tropical glaciers was not considered by Jackson *et al*. (*11*) in their discussion about climate sensitivity of tropical glaciers. Several studies from Kilimanjaro and Rwenzori Mountains emphasize that climate variables related to air moisture (e.g., specific humidity affecting sublimation, cloudiness affecting incoming solar radiation, precipitation affecting glacier surface albedo and mass gain) have a considerable impact on the present surface energy balance of tropical glaciers in Eastern Africa, especially at high elevations above the 0°C isotherm (*22*–*25*). An important question arising from the ongoing debate is to what extent the different climate variables controlled past glacial fluctuations in the region.

If temperature was the primary control on glacier mass balance across Eastern Africa, the local LGM and onset of deglaciation in the region should have occurred rather simultaneously as suggested by Jackson *et al*. (*11*). However, the local LGM in the Ethiopian Highlands predated the global LGM, and deglaciation was not underway before ~18 to 17 ka. While the Mahoma 0 stage (~18 to 17 ka) in the Rwenzori Mountains is located about 5 km up the valley from the Mahoma 1 terminus and clearly indicates pronounced glacier shrinkage after ~20 to 19 ka (*11*), glaciers in the southern Ethiopian Highlands reached almost the same extent at LPG stage II (~19 to 17 ka) as during LPG stage I (~42 to 28 ka). To discuss the potential influence of different climate variables on past glacial fluctuation in Eastern Africa, we compiled additional Late Pleistocene climate proxy records from the region (Fig. 6).

LPG stage I in the Bale Mountains coincided with a climate period favoring positive mass balances and, thus, glacier growth. Temperatures in Eastern Africa decreased continuously from the MIS 4/3 to the MIS 3/2 transition and remained on a relatively low level until ~20 to 18 ka, as proxy data from Lake Tanganyika (6°S; Fig. 1B) in the Great Rift Valley show (Fig. 6). A deuterium record from the same lake indicates furthermore that MIS 3 was generally wetter than MIS 2, despite some distinct drought periods (*26*). The concurrence of relatively low temperatures with a persistent humid period might have triggered the maximum glacier extent in the Bale Mountains before the global LGM. A similar scenario is likely for the High Atlas, which is situated in the precipitation-limited environment of northwestern Africa. The High Atlas is an example for an extratropical climate-glacier system where the local LGM (~75 to 35 ka) coincided with a cold and relatively wet period (*19*, *27*). Examples of a larger glacial extent during MIS 3 than MIS 2 exists also from other mountains worldwide, where precipitation changes controlled spatiotemporal variations in the timing of the local LGM (*28*). Sedimentation rates from the Nile discharge plume in the Mediterranean Sea, lake level fluctuations from Lake Tana (northwestern Ethiopian Highlands; Fig. 1B), and a potassium record from Chew Bahir (southern Ethiopian Rift; Fig. 1B) confirm a change from wetter to drier conditions at the transition between MIS 3 and 2 (*29*–*31*). A decrease in precipitation at that time might have triggered glacial recession in the Bale Mountains at the end of LPG stage I despite continuous low temperatures. However, the large age spread of the local LGM stages in the Bale Mountains hinders the attribution of individual glacier advances to specific climatic events. Furthermore, cold and wet conditions in Eastern Africa should have also triggered glacier expansion in the Rwenzori Mountains during MIS 3. Since the undifferentiated outermost moraines in the Rwenzori Mountains have not yet been dated (*10*–*12*), it can be speculated that they also originate from MIS 3 (or at least are chronostratigraphically older than the Mahoma stage 3 and 4 moraines). During the persistent cold and dry period from ca. 30 to 20 ka, when almost all African Great Lakes were nearly or completely desiccated (*32*, *33*), glaciers in the Bale Mountains were probably inboard of LPG stage II. The contrasting glacier culminations in the Rwenzori Mountains at that time (*10*, *11*) indicate that at least the highest elevations between the Congo Basin and African Great Lakes received enough moisture for sustaining extensive valley glaciers.

The Late Glacial culmination in the Bale Mountains between ~19 and 17 ka might be due to increasingly wetter climatic conditions in the region after ~20 ka as suggested by the potassium record from Chew Bahir (Fig. 6). The glacier culminations in the Bale Mountains versus ongoing glacial recession in the Rwenzori Mountains during that time might be a first hint that paleoglaciers in the Ethiopian Highlands responded sensitively to precipitation changes while those at the equator represented more temperature-driven glacial systems at that time. Modern observations from the Andes support the concept of rather temperature-controlled glaciers in the inner tropics and humidity-controlled glaciers toward the outer tropics (*34*). This shows that tropical glaciers respond to unique forcings based on their specific climatic setting. The onset of deglaciation in the Bale Mountains at 18 to 17 ka and successive glacier recession on Mount Kenya, Kilimanjaro (*9*), and in the Rwenzori Mountains (*12*) toward the end of the Pleistocene is in line with gradually rising temperatures in the period after ~20 ka (Fig. 6). The rising temperatures were accompanied by an upward shift of the ELA, exceeding the maximum elevation of most of the mountains in Eastern Africa.

Besides the timing, also the local LGM ice extent varied among the mountains in Eastern Africa. The ratio of glaciated to total available surface area above the lower ice limits (~3500 m) during the local maximum was much smaller in the Ethiopian Highlands (2 to 29%) compared to the East African Mountains (30 to 80%). This simple comparison ignores variations in the height, relief, and ELA between the different mountains as well as the impact of ice dynamics. In spite of that, the distinct differences in the ratio of glaciated area can be interpreted in terms of a negative precipitation gradient from the equator to the outer tropics (assuming that the ice-covered area decreases if precipitation is limited). Unexpected is the extremely small local LGM extent of only 13 km^2^ in the Simien Mountains (northern Ethiopian Highlands) (*35*), which stands in stark contrast to the existing surface area of 573 km^2^ above 3500 m and 119 km^2^ above 4000 m (Fig. 2). The Simien Mountains are located at the present northern limits of the tropical rain belt (*36*). A slight southward shift of the northern limits of the tropical rain belt during a period of reduced summer insolation on the northern hemisphere might have caused a pronounced drought in the region. Only very dry climatic conditions and the lack of sufficient accumulation basins in this rugged terrain can explain the contrast between minor glaciations in the northern and extensive glaciations in the southern Ethiopian Highlands. However, the climatic interpretation of past glaciations in Eastern Africa remains hypothetical as long as comparative studies on recent afro-alpine precipitation patterns are lacking and discrepancies between latest LGM rainfall simulations and existing paleodata are not resolved (*37*).

The presented glacial chronological and geomorphological work not only provides insights into the climate and glacial history of Eastern Africa but also raises more specific questions regarding the complex plateau glaciation and discrepancy between the ice-free southern escarpment and extensively glaciated northern declivity of the Bale Mountains (Fig. 4). Fluvial erosion along the southern escarpment during the Holocene could theoretically explain the absence of well-preserved glacial deposits, but gullies and other erosion features are lacking. The absence of extensive glaciers in the south could be explained by the lower elevation of the southern plateau and the lack of larger catchments for the accumulation of snow. In addition, prevailing northwesterly to northeasterly winds associated with a distinct precipitation pattern (wet northern and dry southern declivity) might have hindered the formation of glaciers in the south. The phenomenon of larger glaciers (lower ELAs) on windward slopes and smaller glaciers (higher ELAs) in the precipitation shadow is also evident on other African mountains such as Kilimanjaro (*3*, *4*). Periglacial patterns on the southern plateau and relict block fields along the southern escarpment of the Bale Mountains support the idea of a drier southern declivity (fig. S2). Dominating moisture fluxes from north to northeast associated with convection and cloud formation could also explain the lower paleo ELAs in the northern and eastern valleys of the Arsi Mountains (fig. S7) (*3*, *4*). However, note that the prevailing direction of the incoming moisture fluxes to the Ethiopian Highlands does not necessarily indicate where the moisture comes from. Moist air parcels from the Indian Ocean or Congo Basin may flow around the highlands and intrude from the north (*36*, *38*–*40*). To verify whether the hypothesized high-elevation north-south precipitation discrepancy in the Bale Mountains is consistent with present climatic conditions or indicates changes in large-scale circulation patterns during the LGM, further information on the recent synoptic pattern is required.

A peculiarity of the Bale Mountains, which stands out from all other glaciated African mountains except Kilimanjaro, is the formation of an extensive plateau glaciation during the local LGM. Because of equivocal exposure ages and the lack of clear glacial geomorphological features outside the Big Boulder Moraine (Fig. 4), reconstructing the maximum extent and recession stages of the plateau glaciation is fraught with uncertainty. Dating of additional boulders from the inner Big Boulder Moraine and determining the age of glaciofluvial sediments in the northern shallow depressions would help to better constrain the deglaciation history of the plateau. The well-preserved structure and old exposure ages of the stone stripes on the southern and western plateau indicate that not the entire area of the plateau above the reconstructed paleo ELA (~3925 m) was covered by ice (Fig. 4). Ice-free areas above the ELA could be the result of high insolation, variable precipitation, and snowdrift due to strong winds. The tabular ice fields with their characteristic ice walls on Kilimanjaro, which are mainly controlled by solar radiation and changes in precipitation and surface albedo, might serve as a modern analogy for the former plateau glaciation in the Bale Mountains (*41*). The impact of the relief on past ice dynamics (e.g., formation of an ice cap) and precipitation distribution (e.g., north-south gradients) on such scale as the Bale Mountains suggests that besides climatic variations, regional differences in orography also affected the magnitude and rate of glacial fluctuations in the tropical Eastern Africa.

The reconstructed ELA lowering of ~700 m in the Bale Mountains during LPG stage I and the inferred temperature depression of 5.3° ± 0.7°C provides an estimate for the Late Pleistocene temperature decrease and downward shift of the alpine belt in the southern Ethiopian Highlands. Other temperature reconstructions in Eastern Africa from the same period are rare. Proxy data from Lake Rutundu, Lake Tanganyika, and the Congo Basin focus mainly on the global LGM and propose a regional cooling at that time of ~4° to 6°C compared to today (*6*, *26*, *42*). However, because of the steepened lapse rate (*6*) and maximum temperature decrease during MIS 2 (Fig. 6), the temperature and vegetation belt depression in the Ethiopian Highlands was probably even larger during the global LGM than LPG stage I.

Our paleoclimatic and environmental findings from the region have direct implications for the settlement history and ecology of the mountains in Eastern Africa. With our glacial reconstruction, we provide evidence that the repeated residence of Middle Stone Age foragers in the Bale Mountains 47 to 31 cal ka BP (*7*) coincided with a phase of maximum glaciation and up to 6°C cooler temperatures. The large ice-covered area and 700-m downward shift of the alpine belt must have also drastically affected the habitat of endemic species that nowadays populate the central Sanetti Plateau (*14*). Nevertheless, the latest archeological findings (*7*) and high percentage of endemic species in the Bale Mountains (*14*) prove the absence of glacial extinction events in the region. Middle Stone Age foragers as well as local endemic species were coping with the harsh climatic conditions in the Ethiopian Highlands and East African Mountains during Late Pleistocene MIS 3 and MIS 2 periods.

This contribution provides clear evidence that the Ethiopian Highlands were subject to severe climatic and environmental changes during the Late Pleistocene. In the period ~42 to 28 ka, well before the global LGM, glaciers reached their maximum expansion in the Ethiopian Highlands and covered together about 350 km^2^ of the Bale and nearby Arsi Mountains. The mean annual air temperature in higher elevations at that time was 5.3° ± 0.7°C lower than today and caused a downward shift of the afro-alpine belt of about 700 m, reshaping the habitat of endemic species and Middle Stone Age foragers. The glacial chronological comparison reveals that tropical glaciers in Eastern Africa responded at different rates and magnitudes to past climatic changes. The nonuniform glacial fluctuations reflect a regionally varying influence of temperature, precipitation, and orography on glacier mass balance and ice dynamics. While glacial systems in the inner tropics (e.g., Rwenzori Mountains) might have been controlled primarily by temperature, precipitation variations might have played a more important role toward the outer tropics (e.g., Ethiopian Highlands) and in the Mediterranean region (e.g., High Atlas). Our findings highlight the importance of understanding the local climatic setting when attempting to draw wider climatic interpretations from glacial chronologies. To unravel the complex dynamics of the paleoglaciers and paleoclimate in the region, reliable glacial chronologies from the northern Ethiopian Highlands and other equatorial mountains (e.g., Mount Elgon, Aberdare), where moraine sequences are well preserved, are urgently needed.

## MATERIALS AND METHODS

### Terrain analysis

For our terrain analysis of Africa’s alpine environment, we analyzed digital elevation data of the entire continent provided by the CGIAR Consortium for Spatial Information (CGIAR-CSI). The CGIAR-CSI dataset is a void-filled elevation product (*43*, *44*) with a spatial resolution of approximately 93 m at the equator derived from 3–arc sec data (version 2) of the NASA Shuttle Radar Topography Mission (SRTM). As recent studies have shown, SRTM elevation data are suitable for hypsometric and topographic analyses of mountains worldwide owing to the product’s near-global coverage and vertical accuracy (*45*). We downloaded 10 elevation data tiles (each 30° × 30° in size) from the CGIAR-CSI website (version 4.1; available at http://srtm.csi.cgiar.org). The 10 tiles cover together the entire area of the continent. Along the edges, the tiles were overlapping by up to 2 km. We cut and merged the tiles properly to guarantee accurate area-elevation calculations. Since most of the Pleistocene glaciations and periglacial processes in Africa were limited to elevations above 3000 m (*3*, *4*), we neglected areas below this elevation in our analysis. We quantified the continent-wide area above 3000, 3500, and 4000 m using the CGIAR-CSI elevation dataset to specify the extent of the afro-alpine environment. Furthermore, we calculated the surface area distribution with increasing elevation of eight African mountains that were most extensively glaciated during the Pleistocene (*3*, *4*). The relative and absolute surface area based on the CGIAR-CSI dataset was computed for 10-m elevation bands between 3000 m and the individual maximum mountain height.

### Glacial geomorphological mapping

Comprehensive geomorphological mapping of glacial and periglacial features is mandatory for the spatial and chronological reconstruction of past glaciations (*46*). We evaluated maps and photographs of previous studies on Quaternary glaciations in the southern Ethiopian Highlands to compile geomorphological evidence of paleoglaciations in the Bale and Arsi Mountains (*3*, *4*, *14*, *47*, *48*). In contrast to the Arsi Mountains, where moraine sequences are documented in almost every valley (*3*, *4*, *47*), glacial and periglacial features in the Bale Mountains were hitherto only fragmentarily studied on the Sanetti Plateau and in the Wasama, Harcha, and Togona valleys (*7*, *14*, *48*). During our ground reconnaissance, we mapped erratic boulders, moraines, roche moutonnées, and periglacial features all over the Bale Mountains. Furthermore, we analyzed high-resolution DigitalGlobe satellite imagery to identify terminal and lateral moraines in remote areas of the mountain range. If it was feasible, the remote observations were verified in the field later on. High-resolution satellite images were also used to verify and complement previous moraine mapping attempts in the Arsi Mountains (*3*, *4*). For detailed information on the location and characteristics of each mapped moraine in the Bale and Arsi Mountains, see the Supplementary Materials.

### Surface exposure dating

Cosmogenic nuclides commonly used in the context of surface exposure dating such as ^10^Be or ^26^Al are inapplicable in the volcanic Ethiopian Highlands due to the lack of the target mineral quartz in basaltic and trachytic rocks. To develop a robust glacial chronology for the Bale Mountains, we build upon 21 recently published ^36^Cl cosmogenic surface exposure ages (samples HA01 to HA15 and WA01 to WA06) from the Harcha and Wasama valleys (*7*). We analyzed 48 additional erratic boulders (table S2) on stable geomorphic surfaces at 14 different sites in the valleys and on the Sanetti Plateau following established sampling strategies (*49*). Furthermore, we took six rock samples from periglacial features in the south and west of the Sanetti Plateau to determine whether these features have been covered and eroded by thick ice for a longer period. In the southwestern Garemba Valley of the nearby Arsi Mountains, three boulders from the outermost and three from the second innermost moraine were sampled to verify whether the glacial chronology of the Bale Mountains is also representative for other mountains of the southern Ethiopian Highlands. Approximately 1 kg of rock material from the upper 5 cm of each boulder was taken with a hammer, chisel, and angle grinder for subsequent laboratory analysis. An inclinometer was used to measure the topographic shielding.

The 60 rock samples from the Bale and Arsi Mountains were crushed, sieved, and chemically treated in the Surface Exposure Dating Laboratory at the University of Bern. One hundred twenty grams of the 250- to 400-μm grain-size fraction of each whole rock sample was leached in 2 M HNO_3_ and then rinsed with ultrapure water (18.2 megohm·cm) to remove any non–in situ–produced chlorine. From each leached sample, an aliquot of 10 g was sent to Activation Laboratories Ltd. in Ontario, Canada. There, major and trace element concentrations, required for the calculation of the element-dependent local ^36^Cl production rates, were measured (table S3). For the extraction of Cl isotopes, we followed an established protocol based on the method of Stone *et al*. (*50*), using isotope dilution (*51*–*53*). The absence of sufficient phenocrysts in the volcanic samples prevented us from using mineral separates, which can help to reduce the amount of native Cl (*54*) and, therefore, minimize dating uncertainties related to high natural Cl concentrations (*54*, *55*). The leached samples were spiked with a pure ^35^Cl carrier and dissolved using a mixture of HF and HNO_3_. Sulfur was removed from the dissolved samples by precipitating BaSO_3_. Eliminating sulfur from the sample is an essential step, especially in the treatment of volcanic samples, as high concentrations of ^36^S (an isobar of ^36^Cl) can cause interferences during the accelerator mass spectrometry (AMS) measurement. AgNO_3_ was finally added to precipitate AgCl. Total Cl and ^36^Cl concentrations (table S4) were measured from one target at the 6 MV AMS facility of the ETH Zurich based on the isotope dilution technique (*53*) and the gas-filled magnet to separate the remaining ^36^S (*56*). The ETH in-house standard K382/4N (*57*) and the machine blank (natural AgCl) have natural ^37^Cl/^35^Cl ratios and are used to normalize the measured stable isotope ^37^Cl/^35^Cl ratios. The chemistry blank was prepared from a ^35^Cl spike and yielded an isotopic ratio of ^36^Cl/^35^Cl ~ 3 × 10^−15^. In our case, this corresponds to a blank correction of less than 1% for most samples.

We used the measured total Cl and ^36^Cl as well as major and trace element concentrations to calculate surface exposure ages of the boulders from the Bale and Arsi Mountains with the CRONUS Earth Web Calculator version 2.1 (http://cronus.cosmogenicnuclides.rocks/2.1/html/cl/) (*17*). Calibrating ^36^Cl is more difficult and related to larger uncertainties than other widely used cosmogenic nuclides such as ^10^Be or ^26^Al due to the numerous possible target elements (Ca, K, Cl, Ti, and Fe) and different production pathways (spallation, low-energy neutron absorption, and muon capture) (*55*). Site-specific production rates strongly depend on the selected geological calibration dataset and scaling framework. As calibration studies from high elevations in the tropics are generally rare and have not yet been conducted in the Ethiopian Highlands or East African Mountains, it is unclear which production rates and scaling method are best suited for the Bale and Arsi Mountains. We decided to calculate the ^36^Cl surface exposure ages with the calculator by Marrero *et al*. (*17*) using its default production rates and the time-dependent and physics-based LSDn scaling framework (*58*) because it provides satisfying calibration results (*59*), generates nuclide-dependent scaling factors, and accounts for spatiotemporal variations in the geomagnetic and solar magnetic fields. These variations, in turn, affect cosmic-ray fluxes and site-specific production rates, especially at high elevations in the tropics (*17*, *55*, *58*). The LSDn framework is one of the seven scaling models implemented in the web calculator to generate site-specific production rates. All data required by the web calculator are available in a preformatted and ready-to-use spreadsheet (see table S5). To investigate the region-specific exposure age uncertainties related to the choice of the scaling model, we also tested the other six available scaling frameworks. The results of the scaling intercomparison are provided in tables S6 and S7.

We corrected ^36^Cl production rates for sample thickness (provided in table S2), assuming a rock density of 2.65 g cm^−1^ and an attenuation length of 175 g cm^−2^. Correction factors were computed to account for the topographic shielding at the sampling sites (*60*, *61*). The attenuating effect of snow on the local production of ^36^Cl is neglected as snow is rare in the Bale Mountains and melts within hours or days (*14*). During past cold and snowy periods, it would have influenced the total thermal neutron flux, but the related error compared to the other uncertainties is assumed to be relatively small. Postdepositional erosion rates of boulders have not been determined in the Ethiopian Highlands and East African Mountains but are estimated to be in the range of 0 to 2 mm ka^−1^ [a detailed discussion on the effect of erosion on ^36^Cl ages is given in (*9*)]. To account for the impact of erosion, we calculated three different ^36^Cl surface exposure ages for every boulder (see table S4) considering a minimum, medium, and maximum erosion scenario (ε_min_ = 0 mm ka^−1^, ε_med_ = 1 mm ka^−1^, and ε_max_ = 2 mm ka^−1^). The choice of erosion rate in the order of 0 to 2 mm ka^−1^ has a relatively small impact (±0 to 2%) on the calculated exposure age for samples that are younger than ~100 ka but can cause an age variability of more than ±10% for samples older than 100 ka. All exposure ages presented in the text and figures are non-erosion–corrected (ε = 0 mm ka^−1^) and based on the LSDn scaling and default production rates of the applied calculator (*17*). We report the total uncertainty (1σ error) along with each exposure age to enable the direct comparison with other glacial chronologies and climate records. Internal (analytical) errors are provided in table S6 to check the consistency of ages ascribed to a specific glacial stage.

In addition to the exposure ages from the Bale and Arsi Mountains, we considered glacial chronological data from the High Atlas (^10^Be and ^36^Cl) (*19*), Rwenzori Mountains (^10^Be) (*10*–*12*), Mount Kenya, and Kilimanjaro (^36^Cl) (*9*) to study Late Pleistocene glacier fluctuations in tropical Eastern Africa and beyond. A regional comparison requires that exposure ages from different study sites are calculated consistently (i.e., using the same production rates and scaling model) to avoid misinterpretations due to methodological differences. Therefore, we recalculated the original ^10^Be and ^36^Cl data from the High Atlas with the CRONUS Earth Web Calculator using the LSDn scaling scheme. The ^10^Be data from the Rwenzori Mountains are already available with LSDn scaling in the supplementary materials of the original publication (*11*, *12*). It was not possible to reprocess the 20-year-old ^36^Cl data from Mount Kenya and Kilimanjaro (*9*) since only ^36^Cl/Cl and no ^36^Cl concentrations or information on the blank corrections, spiking with Cl carrier, etc. were made available along with the original publication. The recalculated exposure ages from the High Atlas as well as the original data from the Rwenzori Mountains, Mount Kenya, and Kilimanjaro used for Figs. 5 and 6 are provided in tables S8 to S11.

### Glacial chronology

On the basis of the exposure age, location, and geomorphology of the investigated moraines, we distinguished between different glaciations and glacial stages in the Bale Mountains to establish a glacial chronology. The exposure ages of moraine boulders from the upper valleys and Sanetti Plateau allow differentiating between three glacial stages within the Late Pleistocene glaciation (LPG stages I, II, and III). We grouped all exposure ages of moraines associated with the same glacial stage. The Grubbs’ test (*62*) with a significance level of 0.05 was applied to statistically identify outliers in the population of each glacial stage. Moraines comprising three or more inconsistent exposure ages were not classified. The age of each LPG stage is given as arithmetic mean and the uncertainty is given as the SD (1σ) of the population of selected boulders from moraines associated to that glacial stage. An overview of the respective samples used for calculating the mean and SD of each glacial stage is provided in table S7.

### Glacial extent

To assess the potential ice cover extent in the valleys of the Bale Mountains during the local LGM, we reconstructed paleoglacier outlines on the basis of the location of mapped and dated moraines associated with LPG stage I. In those valleys, where no terminal moraines were detected, the elevation of the lower ice limits in the neighboring valleys served as reference for the compilation of the glaciation map. Glacial geomorphological features and landforms, but also outcrops and cliffs, which lack any sign of glacial erosion, were mapped in the head of the upper valleys to delineate the former accumulation areas. In the absence of geomorphological evidence for or against glacial activity, we interpolated the paleoglacier boundaries using the elevation of the lowest glacial landforms from the neighboring valleys as reference.

For the reconstruction of the geometry and extent of the plateau glaciation during LPG stage I, we studied the distribution of erratic boulders around Tullu Dimtu, sediment-filled depressions across the northern plateau, and large periglacial patterns across the southern and western plateau. The boulders encircling Tullu Dimtu were probably deposited after LPG stage I and, therefore, indicate the minimum extent of the former plateau glaciation. Subglacial till and numerous shallow depressions across the northern plateau provide evidence of a much larger plateau glaciation extending down into the western, northern, and eastern valleys. To the south and west of Tullu Dimtu, well-preserved permafrost features exist (figs. S2, C and D, and S3, A and C). Because of the undisturbed structure, barely weathered surface, and substantial ^36^Cl inheritance, we assume that they have not, or at least not for a long time, been covered by thick ice during the local LGM. Therefore, the features serve as markers for the southern and western limit of the plateau glaciation.

The extent of the local LGM glaciation in the Arsi Mountains was reconstructed on the basis of the well-preserved terminal and lateral moraines in the valleys. Since a robust glacial chronology has not yet been established for the Arsi Mountains, ascribing individual moraines to specific glacial periods is not possible. We used the lowest terminal moraines as an approximate indication for the local LGM ice extent. Badly preserved elongated geomorphological structures (mainly below 3600 m) that could not be unequivocally classified as moraines were not considered for the compilation of the glaciation map. In those valleys, where terminal moraines are not preserved, the transition from a broad U-shaped valley to a narrow incised valley was used as a rough indicator for the limits of the local LGM extent. The error introduced into the estimate of the total ice-covered area with regard to the poor age control is relatively small (<10%) as the terminal and lateral moraines from different glacial stages are located very close to each other.

### ELA and temperature reconstruction

The climatic ELA is defined as the average elevation over a 30-year period of a glacier zone where the annual net balance (accumulation versus ablation) is zero (*63*). Since the position of the ELA responds sensitively to climatic changes, it is a useful proxy for temperature reconstructions, especially in alpine regions, where other climate records are often lacking. The most widely used methods for calculating ELAs such as the accumulation area ratio (AAR) or area altitude balance ratio (AABR) require knowledge about the geometry of paleoglaciers (*64*). However, current knowledge about the plateau glaciation in the Bale Mountains is not sufficient to draw contour lines, reconstruct the geometry of the paleo ice cap, and quantify the ice mass turnover from the plateau into the valleys. Hence, we applied a simple THAR, which assumes a constant ratio between the altitude of the frontal position and headwall of the reconstructed paleoglaciers (*64*). A THAR of 0.5 is appropriate for tropical valley glaciers (*48*). We chose three valleys (Harcha, Batu Gudda west, and Batu Gudda east) in the northwest of the Bale Mountains (see Fig. 4) for the ELA reconstruction since these paleoglaciers probably did not gain any mass input from the central ice cap and were fed solely by local snowfall. To back up the calculations, we also determined the MELM in the Harcha, Togona, and eastern Batu Gudda Valley because their upper limit provides a reliable minimum elevation for the paleo ELA. The comparison between paleo and modern ELAs from the same region allows calculating temperature changes relative to today. Because of the absence of present-day glaciers in the Ethiopian Highlands, we used the present mean annual 0°C isotherm (freezing height) as a rough proxy for the modern ELA (*64*, *65*). Hourly temperature data from 1 February 2017 until 31 January 2020 of four recently installed automatic weather stations between 3848 and 4377 m on the Sanetti Plateau served for the computation of an average modern lapse rate (Г_modern_ = 7.0°C km^−1^) and the determination of the present 0°C isotherm (ELA_modern_ ~ 4650 m). For more information, see fig. S6 and its corresponding caption. The temperature depression at LPG stage I (Δ*T* in °C) was calculated using the following formula and assuming a paleo ELA (ELA_paleo_) in the order of 3940 ± 40 m, which reflects the range of reconstructed ELAs (3900 to 3980 m)

$$\Delta T=({\text{ELA}}_{\text{paleo}}-{\text{ELA}}_{\text{modern}})*\Gamma_{\text{paleo}}$$

Since estimates for the local paleo lapse rate (Г_paleo_) in the Ethiopian Highlands are lacking, we use the modern lapse rate (7.0°C km^−1^) from the Sanetti Plateau as a first-order minimum assumption. During MIS 2 (and probably also MIS 3), the lapse rate in the high mountains of tropical Eastern Africa was steeper (ΔГ_paleo-modern_ ~ 1°C km^−1^) than today (most likely due to a drier atmosphere) as reconstructed MIS 2 lake surface temperatures between 474 and 3081 m in the region show (*6*). If we assume a similar lapse rate change (ΔГ_paleo-modern_) for the Bale Mountains, the maximum threshold for the local paleo lapse rate was in the order of 8.0 (Г_modern_ + 1.0) °C km^−1^. To consider the potential range of the paleo lapse rate in the Bale Mountains (7.0° to 8.0°C km^−1^), we applied a paleo lapse rate (Г_paleo_) of 7.5° ± 0.5°C km^−1^ for translating the ELA lowering into a temperature change (Δ*T*).

For calculating the ELA of 19 paleoglaciers in the Arsi Mountains, which were emanating out from the north-south trending ridge, we applied the same procedure (THAR and MELM method) as described above. Since the local LGM in the Arsi Mountains has not yet been determined through surface exposure or radiocarbon dating, it is unclear to which glacial stage the lowest moraines belong. However, the potential error introduced into the ELA estimate is relatively small (<50 m) as the terminal and lateral moraines from different glacial stages are located very close to each other (see fig. S5).

## SUPPLEMENTARY MATERIALS

Supplementary material for this article is available at http://advances.sciencemag.org/cgi/content/full/7/11/eabb6826/DC1
